# Psychological research of the children with chronic kidney disease and their guardians during the COVID-19 pandemic

**DOI:** 10.3389/fpubh.2022.922678

**Published:** 2022-10-17

**Authors:** Hua-Ying Xiong, Gaofu Zhang, Li Wang, Zhijuan Li, Qian Shen, Yuhong Li, Hongtao Zhu, Yue Du, Liangzhong Sun, Bo Zhao, Lijun Zhao, Haidong Fu, Xiaoyan Li, Xiaojie Gao, Sheng Hao, Juanjuan Ding, Zongwen Chen, Zhiquan Xu, Xiaorong Liu, Yuhong Tao, Aihua Zhang, Qiu Li, Mo Wang

**Affiliations:** ^1^Department of Nephrology, Children's Hospital of Chongqing Medical University, Chongqing, China; ^2^Ministry of Education Key Laboratory of Child Development and Disorders, Children's Hospital of Chongqing Medical University, Chongqing, China; ^3^National Clinical Research Center for Child Health and Disorders, Children's Hospital of Chongqing Medical University, Chongqing, China; ^4^China International Science and Technology Cooperation Base of Child Development and Critical Disorders, Children's Hospital of Chongqing Medical University, Chongqing, China; ^5^Chengdu Women's and Children's Central Hospital, School of Medicine, University of Electronic Science and Technology of China, Chengdu, China; ^6^Department of Nephrology, Xi'an Children's Hospital, Xi'an, China; ^7^Department of Nephrology, Children's Hospital of Fudan University, Shanghai, China; ^8^Department of Nephrology, Guiyang Children's Hospital, Guiyang, China; ^9^Department of Pediatrics, The First Affiliated Hospital of Xinjiang Medical University, Ürümqi, China; ^10^Department of Pediatrics, Shengjing Hospital of China Medical University, Shenyang, China; ^11^Department of Pediatrics, Nanfang Hospital, Southern Medical University, Guangzhou, China; ^12^Department of Nephrology, Kunming Children's Hospital, Kunming, China; ^13^Department of Nephrology, Shanxi Children's Hospital, Taiyuan, China; ^14^Department of Nephrology, Children's Hospital, Zhejiang University School of Medicine, Hangzhou, China; ^15^Department of Pediatrics, The Second Xiangya Hospital of Central South University, Changsha, China; ^16^Department of Nephrology, Shenzhen Children's Hospital, Shenzhen, China; ^17^Department of Nephrology and Rheumatology, Shanghai Children's Hospital, Shanghai Jiao Tong University, Shanghai, China; ^18^Wuhan Children's Hospital, Tongji Medical College, Huazhong University of Science and Technology, Wuhan, China; ^19^Chongqing Three Gorges Central Hospital, Chongqing, China; ^20^Department of Nephrology, Rheumatology, and Immunology, Hainan Women and Children's Medical Center, Haikou, China; ^21^Department of Nephrology, Beijing Children's Hospital, Capital Medical University, Beijing, China; ^22^Department of Pediatrics, West China Second University Hospital, Sichuan University, Chengdu, China; ^23^Department of Nephrology, Children's Hospital of Nanjing Medical University, Nanjing, China

**Keywords:** COVID-19, children, chronic kidney disease, anxiety, depression

## Abstract

**Background:**

There is great mental stress due to the coronavirus disease 2019 (COVID-19) pandemic. However, there are no detailed psychological studies of the children with chronic kidney disease (CKD) and their guardians during the COVID-19 pandemic.

**Objective:**

This study explores the psychological pressure on children with CKD and their guardians.

**Methods:**

An online survey was conducted at 20 of the largest pediatric nephropathy departments in China, including the Rutter Parent Questionnaire, Self-rating Anxiety Scale (SAS), and Self-rating Depression Scale (SDS). Overall, 885 children (589 children with CKD associated with 296 children of the control group) completed the survey together with their guardians.

**Results:**

There was no statistical difference between CKD children and control children regarding their Rutter behavior scores and abnormal behaviors. Nevertheless, the abnormal behavior of children might aggravate the anxiety and depression of guardians in both CKD and control groups (*p* < 0.05). We confirmed that the anxiety and depression of guardians in the CKD group were both significantly higher than those in the control group (*p* < 0.05). The guardians in the CKD group with lower annual income were more likely to experience anxiety (*p* < 0.05). Furthermore, the guardians whose children were older than 11 years old might be more anxious than those who were 6–11 years old. Besides, the guardians in the CKD group who watched the news for 30–60 min daily were less likely to have depression than those who watched < 10 min (*p* < 0.05). The subgroup results showed that the gender, the time of watching the news, the annual income of guardians, and children's age might be the most critical factors influencing guardians' psychological burden.

**Conclusion:**

The guardians in the CKD group have more severe anxiety and depression during the pandemic. The children's abnormal behavior, adolescents' pressure, low household income, and the panic about the pandemic may be the main reasons for the anxiety and depression of guardians.

## Introduction

The coronavirus disease 2019 (COVID-19) pandemic, caused by severe acute respiratory disease coronavirus 2 (SARS-CoV-2), has become a global pandemic hence seriously affected people's daily life since December 2019. On the one hand, some threatening variants of SARS-CoV-2 have been reported, including delta and Omicron (1, 2). The pathogenicity and infectivity of these mutant strains are stronger, resulting in an increased prevalence of SARS-CoV-2 in the population. On the other hand, although the vaccine supply is becoming more and more sufficient, the proportion of people vaccinated is increasing, and the effect of the vaccine against mutant strains had waned (3, 4). So far, over 468 million confirmed cases and over 6 million deaths have been reported globally. During this time, the traditional medical pattern has to face the challenges of the COVID-19 pandemic. New medical patterns have been developed, such as internet hospitals and non-contact drug delivery, to help people in need complete medical counseling at home (5). Although the need for medical consultations had been satisfied for patients with chronic diseases, the need for psychological guidance had been often ignored.

In a large-sample, cross-sectional, population-based, online survey study during the COVID-19 pandemic, anxiety was found in 31.6% of the participants and depression in 27.9% of participants, which indicated that mental health symptoms might have been shared among the general population in China (6). Moreover, it was reported in the literature that children with chronic diseases experienced more psychological distress than healthy children, such as chronic lung disease and multiple sclerosis (7, 8). As a chronic disease, CKD requires children to receive long-term medication, regular examinations, and treatment at the hospital. Due to hospitals' cross-infection and low immunity, children with CKD are a high-risk, susceptible group for SARS-CoV-2 (9, 10). The children with CKD are a unique group and may also experience lots of psychological stress.

However, there is no detailed psychological research on children with CKD and their guardians during the COVID-19 pandemic. Our previous investigation showed that the parents of children with CKD might also experience more psychological distress and need more social support compared with those of healthy children (11). Therefore, to further investigate the impact of COVID-19 on the behaviors of children with CKD and the mental stress on their guardians, we conducted this study to help do well in the comprehensive management of CKD children when responding to similar public health incidents in the future.

As far as we know, this is the first study to investigate the behavior conditions in CKD children, and the psychological burden of their guardians during the COVID-19 pandemic. In this study, we revealed the behavioral changes between the CKD and control children, along with the influencing factors of probable anxiety and depression among the guardians of CKD children. It was remarkable that the guardians of CKD children were more likely to experience anxiety and depression than the guardians of control children, indicating that the people's psychological stress should be valued during the pandemic, especially the guardians of CKD children.

## Materials and methods

### Study design and participant recruitment

A cross-sectional network questionnaire survey was carried out at 20 of the largest pediatric nephropathy departments across northeastern, eastern, southern, central, western, and northwestern areas of China from 0:00 on March 1, 2020, to 0:00 on March 12, 2020. Each sampled department is the largest pediatric nephropathy clinic in its province or municipality. The departments reviewed and accepted the questionnaire and delivered it to the guardians, and the questionnaire was recovered and analyzed by the Children's Hospital of Chongqing Medical University.

Children with CKD were diagnosed following the guidelines of KIDGO: abnormalities of kidney structure or function, present for >3 months, with health implications (12). The children of the control group were younger than 18 years old and randomly recruited from the clinics' communities. The exclusion criteria were: 1. children with any chronic disease; 2. had a history of mental illness or family history of mental illness. Before filling out the survey, the researchers explained the study to the participants in a detailed explanation. The questionnaire was delivered to those guardians through a WeChat QR code. If they completed all the questionnaires, it meant that they agreed to participate in this study. The survey was completely anonymous, and the collected data was confidential.

### Questionnaire and data collection

This questionnaire survey had three parts: 1. basic information [address, gender, age, grade in school, duration of illness, identity of the guardian completing the questionnaire, annual household income, and medical treatment (only for children with CKD)]; 2. Guardian Self-rating Anxiety Scale (SAS) and Self-rating Depression Scale (SDS) Questionnaire; 3. Rutter Parent Questionnaire. According to the China Statistical Yearbook (2020), the annual income of 30% of households is <5,000 USD, and the annual income of 60% of the households is <15,000 USD (13). We have set up three levels for families with an annual income of <5,000, 5,000–1,5000 USD, and more than 15,000 USD.

### Measurement standards

Zung's SAS and SDS were used to measure the anxiety and depression of the guardians. As valid tools, these two scales have been widely used to evaluate clinical anxiety and depression, and the reliability and validity of the Chinese version have been confirmed in previous epidemiological surveys (14–16). The SAS and SDS are both self-report scales whose 20 items cover a variety of symptoms. Responses are given on a 4-point scale which ranges from 1 to 4 (1 = rarely, 2 = occasionally, 3 = frequently, 4 = always). Items include both negative and positive experiences, with the latter being reverse scored. The raw scores are converted to index scores by multiplying the sum of the raw scores by 1.25. In the Chinese public, the SAS score has the following four categories: no anxiety (lower than 50), low anxiety (50~59), moderate anxiety (60~69), and severe anxiety (higher than 70). The SDS also has four categories: no depression (lower than 53), low depression (53~62), moderate depression (63~72), and severe depression (higher than 72) (17, 18).

For the Rutter Parent Questionnaire, a total score > 13 indicates that the subject's behavior is abnormal. If the total antisocial behavior (A behavior) score is larger than the neurotic behavior (*N* behavior) score, the subject's behavior should be classified as antisocial; otherwise, it should be classified as neurotic. If the total A behavior score equals the total N behavior score, then the behavior should be classified as mixed behavior (M behavior) (16, 19, 20).

### Statistical analysis

Statistical analyses were conducted in SPSS 26.0 software, and significance was set at *p* < 0.05. Quantitative data conforming to a normal distribution were expressed as the mean ± SD, and data conforming to a non-normal distribution were expressed as the median and quartile. Normal distribution was carried out on continuous variables through the Kolmogorov-Smirnov test, and the Wilcoxon test (Mann-Whitney U test) was adopted for comparison. Categorical variables were tested by Chi-square or Fisher exact test when appropriate. The propensity score matching (PSM) was used to remove confounding bias. Spearman's correlation coefficients were calculated for the correlation analysis. Binary logistic regression analysis was used to identify probable anxiety and depression risk factors.

## Results

### The basic information of CKD children and control children

**Figure 1:**
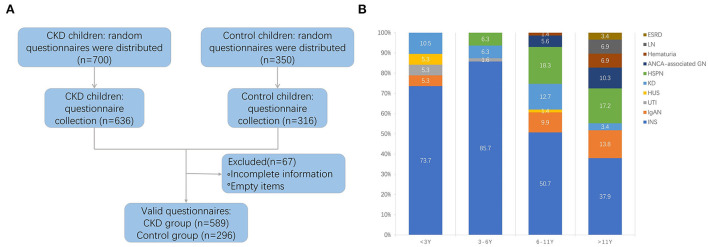
**(A)** Flow diagram of collecting valid questionnaire. CKD, chronic kidney disease. **(B)** The disease spectrum of four age-groups. INS, idiopathic nephrotic syndrome; HSPN, Henoch-Schonlein purpura nephritis; KD, kidney dysplasia; IgAN, IgA nephropathy; ANCA-associated GN, ANCA-associated glomerulonephritis; LN, lupus nephritis; HUS, hemolytic uremic syndrome; UTI, urinary tract infection; ESRD, end-stage renal disease.

Seven hundred questionnaires were provided to the guardians of the CKD group, and 589 valid questionnaires were recovered, with a recovery rate of 84.14%. Three hundred and fifty questionnaires were distributed to the guardians of the control group, and 296 were recovered, with a recovery rate of 84.57% (Figure 1A). To reduce the influence of confounding factors on the study, we performed PSM, and 182 pairs were matched.

The basic information of the two groups is summarized in Table 1. There were no differences in the gender distribution, age, the identity of the questionnaire's respondents, the job and education of guardians, and annual income between CKD and control groups. In our cohort, idiopathic nephrotic syndrome, Henoch-Schonlein purpura nephritis, and kidney dysplasia were the most common diseases, accounting for 84.07%. In addition, there were IgA nephropathy, ANCA-associated glomerulonephritis, lupus nephritis, hemolytic uremic syndrome, hematuria, and urinary tract infection (Supplementary Figure 1). The disease spectrum of four age groups was summarized in Figure 1B.

**Table 1:** Comparison of general information between CKD children and control children.

**Variables**		**CKD (*n*, %)**	**Control (*n*, %)**	**χ^2^**	***P***
Gender	Male	102(56.04)	108 (59.34)	0.41	0.52
	Female	80 (43.96)	74 (40.66)		
Age (Y)	<3	19 (10.44)	25 (13.74)	2.78	0.43
	3–6	63 (34.62)	51 (28.02)		
	6–11	71 (39.01)	80 (43.96)		
	>11	29 (15.93)	26 (14.29)		
Questionnaire respondent	Mother	131 (71.98)	143 (78.57)	2.71	0.26
	Father	48 (26.37)	35 (19.23)		
	Others	3 (1.65)	4 (2.20)		
Job of guardians	Civil servants	16 (8.79)	9 (4.95)	7.37	0.29
	Public institution	50 (27.47)	47 (25.82)		
	Employees	47 (25.82)	55 (30.22)		
	Self-employment venture	24 (13.19)	31 (17.03)		
	Freelancer	29 (15.93)	25 (13.74)		
	Farmer	7 (3.85)	2 (1.10)		
	Others	9 (4.95)	13 (7.14)		
Education of guardians	Middle school	15 (8.24)	13 (7.14)	0.92	0.82
	High school	37 (20.33)	31 (17.03)		
	Junior school	35 (19.23)	38 (20.88)		
	≥Bachelor	95 (52.20)	100 (54.95)		
Annual income (USD)	<5,000	3 (1.65)	7 (3.85)	1.78	0.41
	5,000–15,000	48 (26.37)	50 (27.47)		
	>15,000	131 (71.98)	125 (68.68)		

*CKD, chronic kidney disease*.

During the COVID-19 pandemic, the primary stress for CKD children's guardians was epidemic and basic renal disease (51.65%). 62.09% of guardians in the CKD group believed that the COVID-19 pandemic had affected their child's condition. Meanwhile, most guardians of the CKD group were worried that children were difficult to seek medical care on time and might get infected easier in the pandemic compared to the control group. Online disease counseling, medication, and nutrition counseling were these guardians' most needed medical services (Table 2).

**Table 2:** The basic psychological investigation of guardians of CKD children.

**Basic psychological investigation**	**CKD (*n* = 182) (%)**
**The major stress**	
①COVID-19 pandemic	21 (11.54)
② Basic renal disease	57 (31.32)
③ COVID-19 pandemic + basic renal disease	94 (51.65)
④ Daily life	3 (1.65)
⑤ Family and marriage	1 (0.55)
⑥ Others	6 (3.30)
**The impact of COVID-19 on the condition of children**	
① Few	42 (23.08)
② A little	56 (30.77)
③ Great	57 (31.32)
④ Positive influence	23 (12.64)
⑤ Have no ideas	4 (2.20)
**The probability of coronavirus infection in children with CKD compared with**	
**healthy children**	
① The same	21 (11.54)
② Easier	139 (76.37)
③ Harder	18 (9.89)
④ Have no ideas	4 (2.20)
**The most worrying aspect of CKD children during this pandemic**	
① COVID-19 infection	71 (39.01)
② Unable to see the doctors on time	93 (51.10)
③ Children can't go out and study normally	17 (9.34)
④ Others	1 (0.55)
**The help need most for the guardians**	
① Online renal disease counseling	108 (59.34)
② Medication and nutrition guidance	68 (37.36)
③ Psychological counseling	1 (0.55)
④ Others	5 (2.75)

### Comparison of the rutter behavior scores between CKD children and control children

**Figure 2:**
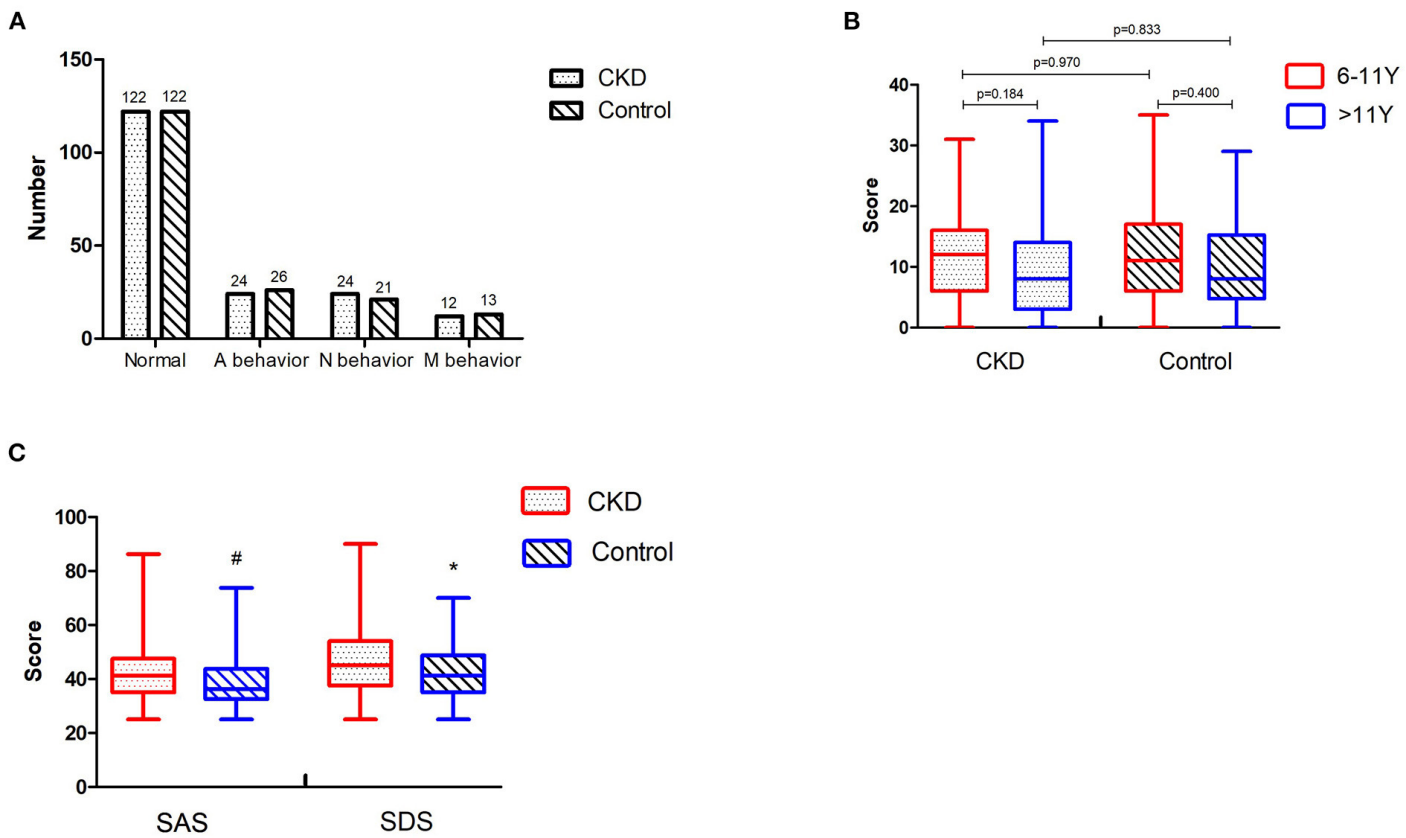
**(A)** Comparison of Rutter behaviors between CKD children and control children. **(B)** Comparisons of Rutter behavior scores at different ages within the same group, and comparisons at the same age between different groups. **(C)** Comparisons of anxiety and depression between the guardians in the CKD group and the control group. CKD, chronic kidney disease; A behavior, antisocial behavior; N behavior, neurotic behavior; M behavior, mixed behavior; “#” means *p* < 0.001; “*” means *p* < 0.05.

The Rutter behavior scores of CKD children and control children were 11.00 (5.75–16.00) and 10.00 (5.00–16.00), respectively; the difference was not significant (*p* = 0.65). The two groups of children had similar frequencies of antisocial behavior (A behavior) and neurotic behavior (*N* behavior), as well as mixed behavior (M behavior), with no significant differences (χ^2^ = 0.32, *p* = 0.85) (Figure 2A).

We conducted more studies in different age groups to better understand the relationship between children's age and Rutter behavior scores. Because the child behavior scores from the Rutter Parent Questionnaire are more applicable for children older than 5 years old, the children were divided into two groups, including 6–11 years old (6–11 y) and older than 11 years old (>11 y). No significant differences between CKD and control groups were observed in the Rutter behavior scores in either 6–11 y or >11 y groups (*p* > 0.05). There was no difference between different age groups in CKD or control group (*p* > 0.05) (Figure 2B).

### Comparison of the SAS and SDS scores between the guardians of CKD children and control children

The SAS and SDS scores of guardians in the CKD group were 41.25 (35.00–47.50) and 45.00 (37.50–54.06), respectively, while the scores of guardians in the control group were 36.25 (32.50–43.75) and 41.25 (35.00–48.75). There were 17 (9.34%), 12 (6.59%), and 4 (2.20%) guardians in the CKD group who displayed mild, moderate, and severe anxiety, respectively, indicating that many guardians in the CKD group experienced anxiety. There were 14 (7.69%), 3 (1.65%), and 1 (0.55%) guardians in the control group who displayed mild, moderate, and severe anxiety, respectively, indicating that a few guardians in the control group experienced mild anxiety. The SAS scores of guardians in the CKD group were markedly higher than those in the control group (Z = −3.96, *p* < 0.001).

There were 35 (19.23%), 14 (7.69%), and 3 (1.65%) guardians in the CKD group who displayed mild, moderate, and severe depression, respectively, indicating that about a third of the guardians in the CKD group experienced depression. There were 19 (10.44%), 13 (7.14%), and 0 (0) guardians in the control group who displayed mild, moderate, and severe depression, respectively, indicating that a few guardians in the control group experienced mild depression. The SDS scores were significantly higher among the guardians in the CKD group than those in the control group (Z = −2.68, *p* = 0.01) (Figure 2C).

In addition, we used Spearman's correlation analysis to identify the relationship between the Rutter behavior scores of children and the mental stress of guardians. The results showed that the Rutter behavior scores were correlated with SAS and SDS in both CKD and control groups (*p* < 0.05). Though the correlations in the two groups were not very strong, they still indicated that the children's abnormal behaviors might aggravate the anxiety and depression of guardians (Table 3).

**Table 3:** Correlation analysis of the scores of children and their guardians.

		**Spearman's rho**	***p***
**CKD group**
Rutter vs. SAS		0.19	0.01
Rutter vs. SDS		0.28	<0.01
**Control group**
Rutter vs. SAS		0.24	<0.01
Rutter vs. SDS		0.26	<0.01

*CKD, chronic kidney disease*.

### Analysis of factors influencing anxiety and depression of guardians

We used Chi-squared Test and Binary logistic regression analysis to identify the CKD group's probable anxiety and depression risk factors in further analysis. Variables showing *p* < 0.1 in the Chi-squared test were selected for entry into the Binary logistic regression analysis. The Chi-squared test results showed that the age, grade and disease duration of children, and annual household income were likely to be the risk factors of anxiety in the guardians of the CKD group (Supplementary Table 1). Additionally, the gender of children, education, and the time of watching the news of guardians might be the risk factors for depression in the guardians of the CKD group (Supplementary Table 2).

The results of the Binary logistic analysis indicated that compared with the group of children older than 11 years old group, the guardians whose children were in the 6–11 y had a lower risk of anxiety (OR = 0.16, *p* = 0.02). When compared with the guardians earning more than 15,000 USD, the guardians earning <5,000 USD were more likely to have anxiety (OR = 20.96, *p* = 0.03) (Table 3). Moreover, compared with the CKD guardians who watched the news <10 min daily, the guardians who watched the news for 30–60 min daily were less likely to have depression (OR = 0.24, *p* = 0.01) (Tables 4, 5).

**Table 4:** Binary logistic regression analysis of risk factors of anxiety in guardians of CKD children.

**Overall**			**Female** ^**†**^			>**15,000 USD**			<**6 y**		
**Variables**	**OR (95%CI)**	***P***	**Variables**	**OR (95%CI)**	***P***	**Variables**	**OR (95%CI)**	***P***	**Variables**	**OR (95%CI)**	***P***
Age			Age			Age			Gender		
>11 y	1.00		>11 y	1.00		>11 y	1.00		Male	1.00	
<3 y	1.32 (0.14–12.72)	0.81	<3 y	1.62 (0.37–7.09)	0.52	<3 y	0.7 (0.15–3.17)	0.64	Female	3.83 (1.11–13.28)	0.03
3–6 y	0.45 (0.06–3.40)	0.44	3–6 y	0.49 (0.13–1.82)	0.29	3–6y	0.21 (0.06–0.73)	0.02			
6–11 y	0.16 (0.03–0.78)	0.02	6–11 y	0.19 (0.04–0.80)	0.02	6–11y	0.11 (0.02–0.47)	<0.01			
AHI			AHI								
>15,000	1.00		>15,000	1.00							
<5,000	20.96(1.35–324.91)	0.03	<5,000	22.84 (1.51–344.81)	0.02						
5,000–15,000	1.62 (0.63–4.15)	0.31	5,000–15,000	2.43 (0.87–6.82)	0.09						

*CKD, chronic kidney disease; AHI, Annual household income. “*^†^”* means that in the subgroup analysis of gender, the total number of CKD guardians was 181, because the gender of one guardian was unknown*.

**Table 5:** Binary logistic regression analysis of risk factors of depression in guardians of CKD children.

**Overall**			<**15,000 USD**			<**6 y**			>**6 y**		
**Variables**	**OR (95%CI)**	***P***	**Variables**	**OR (95%CI)**	***P***	**Variables**	**OR (95%CI)**	***P***	**Variables**	**OR (95%CI)**	***P***
Time of watching the news			Time of watching the news			Gender			Time of watching the news		
<10 min	1.00		<10 min	1.00		Male	1.00		<10 min	1.00	
10–30 min	0.34 (0.11–1.02)	0.05	10–30 min	0.28 (0.06–1.41)	0.12	Female	3.73 (1.27–10.98)	0.02	10–30 min	0.13 (0.02–0.77)	0.02
30–60 min	0.24 (0.08–0.73)	0.01	30–60 min	0.13 (0.02–0.72)	0.02				30–60 min	0.05 (0.01–0.33)	<0.01
1–3 h	0.41 (0.12–1.45)	0.17	1–3 h	0.00	1.00				1–3 h	0.17 (0.03–1.09)	0.06
>3 h	0.10 (0.01–1.05)	0.06	>3 h	0.00	1.00				>3 h	0.05 (0.003–0.67)	0.02

CKD, *chronic kidney disease*.

In addition, based on the overall analysis results and previous studies, we had further performed the subgroup analysis of gender, annual household income of guardians, and children's age. In the female guardians subgroup, the Binary logistic regression analysis results showed that the guardians whose children were 6–11 y had a lower risk of anxiety than the >11 y group (OR = 0.19, *p* = 0.02). In addition, the guardians earning <5,000 USD were more likely to have anxiety than those over 15,000 USD (OR = 22.84, *p* = 0.02) (Table 4). There were no risk factors for anxiety or depression in the male group. In the annual income subgroup of earning more than 15,000 USD, compared with the oldest group, the guardians whose children were 3–6 years old had a lower risk of anxiety (OR = 0.21, *p* = 0.02), as well as the group 6–11 y (OR = 0.11, *p* = < 0.01) (Table 4). In the subgroup earning <15,000 USD, the guardians who watched the news for 30–60 min were less likely to have depression (OR = 0.13, *p* = 0.02) (Table 5). In the subgroup younger than 6 years old, the female guardians had a higher risk of anxiety (OR = 3.83, *p* = 0.03) and depression (OR = 3.73, *p* = 0.02) than males (Tables 4, 5). In the subgroup older than 6 years old, the guardians who watched the news for 10–30, 30–60 min, or >3 h were less likely to have depression than those who watched <10 min (*p* < 0.05) (Table 5).

## Discussion

CKD is a common chronic disease, and COVID-19 significantly threatens the life of immunocompromised patients with CKD, especially children. Many studies reported that CKD was one of the independent factors associated with death during the COVID-19 pandemic (9, 21). In this condition, the guardians of CKD children are prone to experience heavier mental pressure. Our previous investigation showed that the guardians of CKD children had worse social support and greater loneliness than guardians in healthy group (11). We got a high recovery rate of questionnaires in our study. One reason was that the questionnaire was delivered by every sampled department. The guardians in the CKD group were quite cooperative due to the favorable treatment compliance during the children's long-term follow-up. Another reason was that ordinary people were concerned about the epidemic during the COVID-19 pandemic.

Our study revealed that guardians of CKD children were more likely to have anxiety and depression, which might have been related to the primary disease itself, the higher susceptibility of CKD children to COVID-19, and inconvenient medical treatment. Most guardians of CKD children were worried that their children might not be able to seek medical care promptly; at the same time, they were also concerned about the risk of infection in medical institutions (22). Under such tremendous pressure, guardians may experience sadness, depression, helplessness, irritability, and anxiety. Fortunately, the hospitals have introduced a series of measures, including online consultation, “Internet +” hospitals, non-contact drug delivery, and online psychological counseling, which may effectively relieve these families' medical and psychological pressure (5, 23).

CKD children are a high-risk group during the epidemic and the vulnerable group during a stressful event; they are more likely to feel fear and worry about the epidemic (9, 10). On the one hand, excessive parental attention and anxiety about their children may lead to children's abnormal behavior. On the other hand, our study indicated that the children's abnormal behavior might also aggravate the anxiety and depression of the guardians, which was similar to the study of Korean children (24). Therefore, the guardians need to correctly identify and deal with the stress responses of their children during the COVID-19 pandemic (25, 26). The Subspecialty Group of Child Health Care of the Chinese Medical Doctor Association had provided many intervention suggestions for the children and their families at the beginning of the epidemic, which may be helpful. The guardians are supposed to maintain their children's composure and help them feel loved in the epidemic. The children will be relieved if they acknowledged the constant protection and accompaniment by adults or peers (27). Children and their parents may need psychological counseling or professional intervention if necessary.

The annual income and economic pressure of the whole family may remarkably influence the mental state of guardians. The guardians with a lower household income are much more likely to suffer from anxiety than higher income, so the higher income was a protective factor against anxiety. Thus, we supposed that the mental pressure of the guardians in the CKD group might also be alleviated with the excellent control of the epidemic and the reduction of family economic pressure (28, 29). Besides, compared with the group of children older than 11 years old, the guardians whose children were 6–11 years old had a lower risk of anxiety. The primary diseases of children might influence the anxiety of the guardian, so we performed a Chi-square test of the disease spectrum to compare the difference between the 6–11 and >11 y group, which turned out to be no different. However, a population-based repeated cross-sectional study reported that adolescents increased mood and anxiety disorders during the COVID-19 pandemic (30). The prospective longitudinal cohort study from the USA found that older children aged 12 to 15 years reported greater perceived stress (31). We supposed that adolescents' stress could be the main reason for more anxiety among the guardians in the >11 y group.

In addition, we found that CKD guardians watching the COVID-19-related news for 30–60 min daily were less expected to have depression, compared with the guardians for <10 min. It had been reported that participants with low knowledge of COVID-19 were more likely to experience mental health and more substance use (32). Besides, increased time spent on social media and consulting more traditional media sources about COVID-19 had been confirmed independently associated with increased mental distress (33). We speculated that if the guardians spent more time focusing on the epidemic and received more information, their depression would be relieved. Alternatively, if they spent considerable amount of time on COVID-19, it could also reflect their severe mental pressure. In general, the government should disclose positive information about the epidemic as much as possible, which will help alleviate people's depression during the epidemic. In addition, the subgroup analysis results also revealed that female guardians, low annual income, older children, and lack of understanding of COVID-19 might be the essential factors aggravating guardians' psychological burden.

Our study had several limitations. First, we used the SAS and SDS to evaluate the levels of anxiety and depression but didn't include severe psychiatric symptoms, such as suicidal ideation. Second, the network questionnaire was taken and well-accepted mainly by young people, which might cause sampling bias to some extent (34). Third, due to the home quarantine awareness and limited time for collecting questionnaires, the sample size of the control group in our study was smaller than the CKD group. Fourth, although guardians in the ESRD group may experience more mental stress, we did not subdivide the CKD group according to renal function because the original questionnaire had no information on renal function, and it was almost impossible to repeat the questionnaire with the same anonymous study population.

The guardians in the CKD group have more severe anxiety and depression during the pandemic than the control group. The guardians of lower-income families are more likely to experience anxiety, and more social support is needed for these children and their families. In addition, the guardians with high knowledge of pandemics are less likely to experience depression, indicating that prompt and accurate information guidance may help relieve psychological stress. The guardians whose children were older than 11 years old experienced more anxiety, showing that the psychological pressure of adolescents and their guardians should be fully valued. Therefore, under the pandemic circumstance, the government shall pay more attention to the psychological stress of patients with chronic diseases and their guardians, disclose the epidemic-related information as soon as possible, and provide more access to psychological counseling for those in need.

## Conclusion

In summary, our study focused on the unique group, CKD children and their guardians, which might be ignored during the COVID-19 pandemic. Compared with the control group, the guardians in the CKD group were more likely to have anxiety and depression during the pandemic. Female guardians, low annual income, older children, and lack of understanding of COVID-19 might be the most critical factors aggravating guardians' psychological burden on the CKD group. The psychological health of patients with chronic diseases and their guardians should be valued, and the government should provide various social support, which may help improve the comprehensive management of CKD children. The change in environment (such as the management of epidemic, and vaccination) may relieve the mental stress of children and guardians, but their psychological stress should still be concerned.

## Data availability statement

The raw data supporting the conclusions of this article will be made available by the authors, without undue reservation.

## Ethics statement

The studies involving human participants were reviewed and approved by the Ethics Committee of Children's Hospital of Chongqing Medical University. Written informed consent from the participants' legal guardian/next of kin was not required to participate in this study in accordance with the national legislation and the institutional requirements.

## Author contributions

Research idea and study design: MW, AZ, and QL. Data acquisition: MW, H-YX, GZ, LW, ZL, QS, YL, HZ, YD, LS, BZ, LZ, HF, XLi, XG, SH, JD, ZC, ZX, XLiu, and YT. Data analysis/interpretation and statistical analysis: MW, H-YX, and GZ. All authors contributed important intellectual content during manuscript drafting or revision and accepted accountability for the overall work by ensuring that questions about the accuracy or integrity of any portion of the work were appropriately investigated and resolved, contributed to the article, and approved the submitted version.

## Funding

This study was sponsored by Research Program of Innovative Research Demonstration Base of Children's Medical Security (Grant No: NCRCCHD-2019-HP-09), the COVID-19 Emergency Research Project of Chongqing Medical University (CQMUNCP0310), and National Clinical Research Center for Child Health and Disorders (Grant No: NCRC-2020-GP-01).

## Conflict of interest

The authors declare that the research was conducted in the absence of any commercial or financial relationships that could be construed as a potential conflict of interest.

## Publisher's note

All claims expressed in this article are solely those of the authors and do not necessarily represent those of their affiliated organizations, or those of the publisher, the editors and the reviewers. Any product that may be evaluated in this article, or claim that may be made by its manufacturer, is not guaranteed or endorsed by the publisher.
