# Supplementary material for: Psychological research of the children with chronic kidney disease and their guardians during the COVID-19 pandemic
Source: Front Public Health. 2022 Oct 17;10:922678. doi: 10.3389/fpubh.2022.922678 (PMC9621117; doi:10.3389/fpubh.2022.922678)
Supplement: Supplementary file 1 [file Table_1.DOCX]

Table 1 Analysis of the influencing factors of anxiety in guardians of CKD group (SAS)

| Variables | CKD | | **χ^2^** | *P* |
| --- | --- | --- | --- | --- |
|  | Normal(n=149) | Anxiety(n=33) |  |  |
| Children’s gender | | | | |
| male | 86(57.7) | 16(48.5) | 0.935 | 0.334 |
| female | 63(42.3) | 17(51.5) |  |  |
| Children’s age(Y) | | | | |
| <3 | 13(8.7) | 6(18.2) | 9.876 | 0.017 |
| 3-6 | 54(36.2) | 9(27.3) |  |  |
| 6-11 | 63(42.3) | 8(24.2) |  |  |
| >11 | 19(12.8) | 10(30.3) |  |  |
| Disease duration | | | | |
| <3m | 7(4.7) | 2(6.1) | 8.287 | 0.066 |
| 3-6m | 15(10.1) | 0 |  |  |
| 6-12m | 16(10.7) | 8(24.2) |  |  |
| 1-3y | 70(47.0) | 12(36.4) |  |  |
| >3y | 41(27.5) | 11(33.3) |  |  |
| Children’s grade | | | | |
| Kindergarten | 46(30.9) | 6(18.2) | 8.678 | 0.091 |
| Primary school | 62(41.6) | 12(36.4) |  |  |
| Middle school | 8(5.4) | 6(18.2) |  |  |
| High school | 6(4.0) | 1(3.0) |  |  |
| University | 0 | 0 |  |  |
| Quit school | 24(16.1) | 6(18.2) |  |  |
| Others | 3(2.0) | 2(6.1) |  |  |
| Respondent | | | | |
| Mother | 107(71.8) | 24(72.7) | 0.242 | 1.00 |
| Father | 39(26.2) | 9(27.3) |  |  |
| Others | 3(2.0) | 0 |  |  |
| Education | | | | |
| middle school | 11(7.4) | 4(12.1) | 2.416 | 0.484 |
| High school | 33(22.1) | 4(12.1) |  |  |
| junior college | 29(19.5) | 6(18.2) |  |  |
| bachelor | 76(51.0) | 19(57.6) |  |  |
| Annual income (USD) | | | | |
| <5000 | 1(0.7) | 2(6.1) | 5.512 | 0.045 |
| 5000-15000 | 37(24.8) | 11(33.3) |  |  |
| >15000 | 111(74.5) | 20(60.6) |  |  |
| Time to watch the news | | | | |
| <10min | 15(10.1) | 5(15.2) | 2.472 | 0.659 |
| 10-30min | 46(30.9) | 7(21.2) |  |  |
| 30-60min | 55 (36.9) | 14(42.4) |  |  |
| 1-3h | 27(18.1) | 5(15.2) |  |  |
| >3h | 8(4.0) | 2 (6.1) |  |  |
| Job | | | | |
| civil servants | 12(8.1) | 4(12.1) | 5.214 | 0.498 |
| Public institution | 38(25.5) | 12(36.4) |  |  |
| employees | 40(26.8) | 7(21.2) |  |  |
| Self-employment venture | 19(12.8) | 5(15.2) |  |  |
| freelancer | 27(18.1) | 2(6.1) |  |  |
| farmer | 6(4.0) | 1(3.0) |  |  |
| others | 7 (4.7) | 2(6.1) |  |  |

Table 2 Analysis of the influencing factors of depression in guardians of CKD group (SDS)

| Variables | CKD | | **χ^2^** | *P* |
| --- | --- | --- | --- | --- |
|  | Normal（n=130） | Depression（n=52） |  |  |
| Children’s gender | | | | |
| male | 78(60.0) | 24(46.2) | 2.891 | 0.089 |
| female | 52(40.0) | 28(53.8) |  |  |
| Children’s age(Y) | | | | |
| <3 | 11(8.5) | 8(15.4) | 3.469 | 0.325 |
| 3-6 | 45(34.6) | 18(34.6) |  |  |
| 6-11 | 55(42.3) | 16(30.8) |  |  |
| >11 | 19(14.6) | 10(19.2) |  |  |
| Disease duration | | | | |
| <3m | 7(5.4) | 2(3.8) | 2.834 | 0.593 |
| 3-6m | 13(10.0) | 2(3.8) |  |  |
| 6-12m | 15(11.5) | 9(17.3) |  |  |
| 1-3y | 59(45.4) | 23(44.2) |  |  |
| >3y | 36(27.7) | 16(30.8) |  |  |
| Children’s grade | | | | |
| kindergarten | 36(27.7) | 16(30.8) | 7.198 | 0.186 |
| primary school | 57(43.8) | 17(32.7) |  |  |
| middle school | 7 (5.4) | 7(13.5) |  |  |
| high school | 6(4.6) | 1(1.9) |  |  |
| university | 0 | 0 |  |  |
| quit school | 22(16.9) | 8(15.4) |  |  |
| others | 2(1.5) | 3(5.8) |  |  |
| Respondent | | | | |
| Mother | 91(70.0) | 40(76.9) | 3.728 | 0.128 |
| Father | 38(29.2) | 10(19.2) |  |  |
| Others | 1(0.8) | 2(3.8) |  |  |
| Education | | | | |
| middle school or below | 7(5.4) | 8(15.4) | 6.409 | 0.091 |
| High school | 24(18.5) | 13(25.0) |  |  |
| junior college | 27(20.8) | 8(15.4) |  |  |
| Bachelor degree or above | 72(55.4) | 23(44.2) |  |  |
| Annual income (USD) | | | | |
| <5000 | 2(1.5) | 1(1.9) | 0.591 | 0.872 |
| 5000-15000 | 33(25.4) | 15(28.8) |  |  |
| >15000 | 95(73.1) | 36(69.2) |  |  |
| Time to watch the news | | | | |
| <10min | 9(6.9) | 11(21.2) | 8.658 | 0.063 |
| 10-30min | 37(28.5) | 16(30.8) |  |  |
| 30-60min | 54(41.5) | 15(28.8) |  |  |
| 1-3h | 23(17.7) | 9(17.3) |  |  |
| >3h | 7(5.4) | 1(1.9) |  |  |
| Job | | | | |
| civil servants | 12(9.2) | 4(7.7) | 4.283 | 0.648 |
| public institution | 34(26.2) | 16(30.8) |  |  |
| employees | 37(28.5) | 10(19.2) |  |  |
| self-employment venture | 16(12.3) | 8(15.4) |  |  |
| freelancer | 22(16.9) | 7(13.5) |  |  |
| farmer | 4(3.1) | 3(5.8) |  |  |
| others | 5(3.8) | 4(7.7) |  |  |


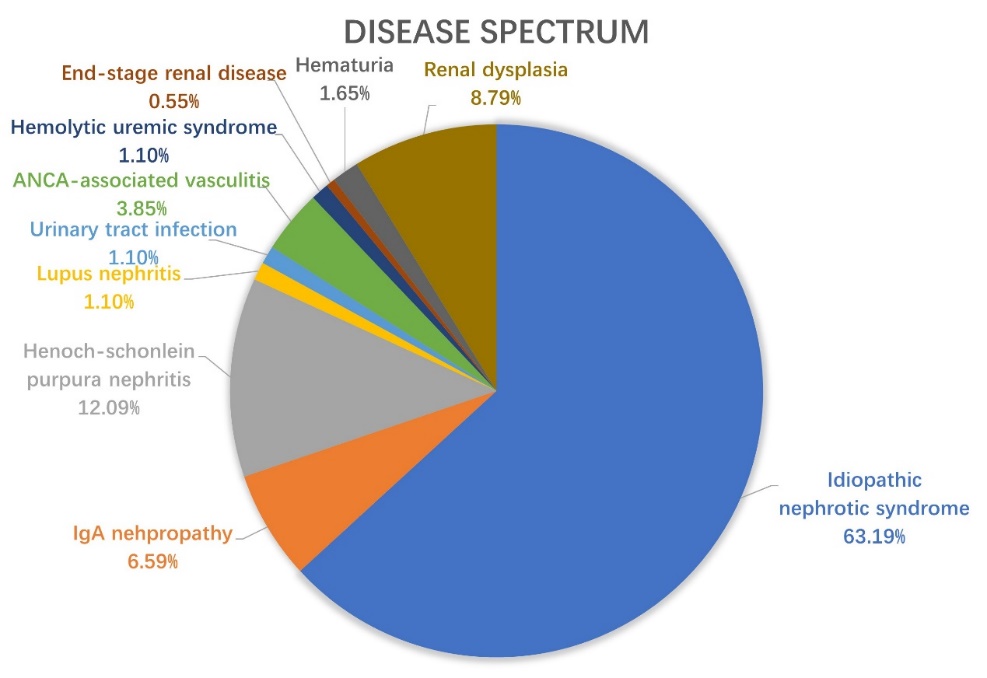


Figure 1. The disease spectrum in our cohort.
